# Low-Cost Impedance Camera for Cell Distribution Monitoring

**DOI:** 10.3390/bios13020281

**Published:** 2023-02-16

**Authors:** Bo Tang, Mengxi Liu, Andreas Dietzel

**Affiliations:** 1 Institute of Microtechnology (IMT), Technische Universität Brauschweig, Alte Salzdahlumer Str. 203, 38124 Brauschweig, Germany; 2 Center of Pharmaceutical Engineering (PVZ), Technische Universitãt Braunschweig, Franz-Liszt-Straße 35a, 38106 Braunschweig, Germany

**Keywords:** electrical impedance spectroscopy (EIS), impedance camera, yeast cell suspension, microelectrode array (MEA), printed circuit board (PCB), bioimaging

## Abstract

Electrical impedance spectroscopy (EIS) is widely recognized as a powerful tool in biomedical research. For example, it allows detection and monitoring of diseases, measuring of cell density in bioreactors, and characterizing the permeability of tight junctions in barrier-forming tissue models. However, with single-channel measurement systems, only integral information is obtained without spatial resolution. Here we present a low-cost multichannel impedance measurement set-up capable of mapping cell distributions in a fluidic environment by using a microelectrode array (MEA) realized in 4-level printed circuit board (PCB) technology including layers for shielding, interconnections, and microelectrodes. The array of 8 × 8 gold microelectrode pairs was connected to home-built electric circuitry consisting of commercial components such as programmable multiplexers and an analog front-end module which allows the acquisition and processing of electrical impedances. For a proof-of-concept, the MEA was wetted in a 3D printed reservoir into which yeast cells were locally injected. Impedance maps were recorded at 200 kHz which correlate well with the optical images showing the yeast cell distribution in the reservoir. Blurring from parasitic currents slightly disturbing the impedance maps could be eliminated by deconvolution using an experimentally determined point spread function. The MEA of the impedance camera can in future be further miniaturized and integrated into cell cultivation and perfusion systems such as organ on chip devices to augment or even replace light microscopic monitoring of cell monolayer confluence and integrity during the cultivation in incubation chambers.

## 1. Introduction

A broad range of bioimaging tools have been developed to provide valuable biological information and improve the reproducibility of cell cultivations [1,2,3,4]. In both, suspended and adherent cell cultures, homogenous distributions of cells over a certain volume or surface area are a key for good growth, uniform cell environment, and reproducible experiments or tests [5,6]. The widely used optical digital imaging systems are typically bulky with complex optical lenses and a charge-coupled device (CCD), which is in the way of continuous monitoring of cell cultures and micro-perfusion systems placed in incubators. Moreover, prolonged light irradiation potentially affects the replicative lifespan of the cells [7]. Monitoring the cell density without imaging by plate counting, spectrophotometry, or flow cytometry is not only costly but also requires a disruption of the cultivation process. Innocuous ultrasound for the characterization of biological tissues presents as a suitable tool for the label-free measurement of cell concentration [8]. However, ultrasound sensing of suspended cells requires high frequency ultrasound pulses around 50 MHz [8,9], which makes a system too complex as far as on-chip recognition is concerned.

Light-addressable potentiometric sensors can sense not only the electrical properties of cells but also the metabolic activity of cells in a spatially resolved manner [10,11]. With LEDs or laser beams, one can map the surface with high spatial resolution [12]. However, when cells are adherent to a freestanding ultrathin porous membrane as is the typical configuration of organ on chip devices for drug permeation studies [13,14], this concept is not applicable. In contrast to the named methods, electrical characterization is an attractive technique because microelectrodes can easy be integrated in membranes. Moreover, electrical signals are both easy to generate on a chip and easy to process digitally. The electrical impedances measured in cultures with suspended or adherent cells contain contributions from ohmic resistances and capacities which usually have specific physical and biological meanings [15]. A variety of electrical impedance imaging platforms have been developed over the last few decades and are becoming essential in the field of bioimaging of cells and tissues [2]. Electrochemical scanning generates high-resolution images of cell spheroids and cell membranes but requires profound electrochemical interpretation and comes with the drawback of being time-consuming. On-chip electrochemical systems were developed with either single electrodes or electrode arrays, which were realized with clean room fabrication and expensive material deposition/etching techniques. The relatively new technique of electrical impedance tomography (EIT) is based on small currents injected into the cell cultures by electrodes surrounding the sample and reconstruction of 3D spatial shapes of cell distributions from measured signals. High spatial resolution of EIT requires very small electrodes, suitable current injection strategies, and complex mathematical image reconstruction algorithms. EIT studies on cells have so far been focused on imaging 3D cell spheroids and cell aggregates [16], but to our knowledge not on suspended cells and monolayers on cell membranes.

The high-frequency electrical properties of particle or cell suspensions depend on their size and volume fraction [17].

**Figure 1:**
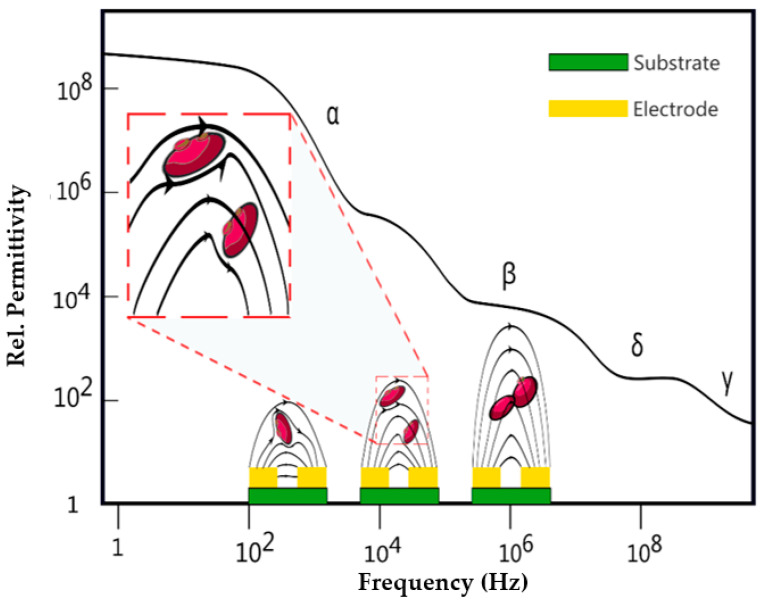
The general course of permittivity of a cell suspension in dependence of frequency with a schematic illustration of the field line penetration corresponding to the regions of α and β beta dispersion and the transition between the two.

For cells in suspension, four frequency regions with different relative permittivity can be distinguished (Figure 1) [3]: the α-dispersion in range of 100 Hz to 10 kHz corresponds to ion diffusion along cell-to-cell interfaces and through the solution in which cells are suspended; β-dispersion in range of sub-MHz and 10 MHz corresponds to polarization at the cellular membrane which allows the electrical field lines passing through the cells. δ and γ –dispersion is observed in sub-GHz and over 10 GHz frequencies and correspond to protein properties which are not in the scope of our work. In general, for a given geometry of coplanar electrodes, the field penetration depth (in z-direction assuming x/y directions are in the plane of the electrodes) can depend on the excitation frequency [18,19] and therefore the depth within the material from which information can be obtained. For co-planar thin film electrodes, the larger the electrode surface area and the smaller the electrode spacing, the smaller the measured impedance. For adherent cells, a closer spaced electrode arrangement is advantageous for measuring cell information between the two electrodes, while for cells in suspension, a wider electrode spacing is advantageous because the electric field extends further in the z-direction. Arrayed co-planer electrodes even provide spatial information with a resolution depending on the size and pitch of electrode pairs.

In this work, simple two-electrode measurement areas, which can be placed spatially densely, were arranged as an 8 × 8 array based on a commercial PCB technology to establish a low-cost impedance camera system. A low-cost impedance readout electronics based on a microcontroller and two multiplexers addressing the electrode array was developed. 

## 2. Materials and Methods

### 2.1. Impedance Imaging Array

**Figure 2:**
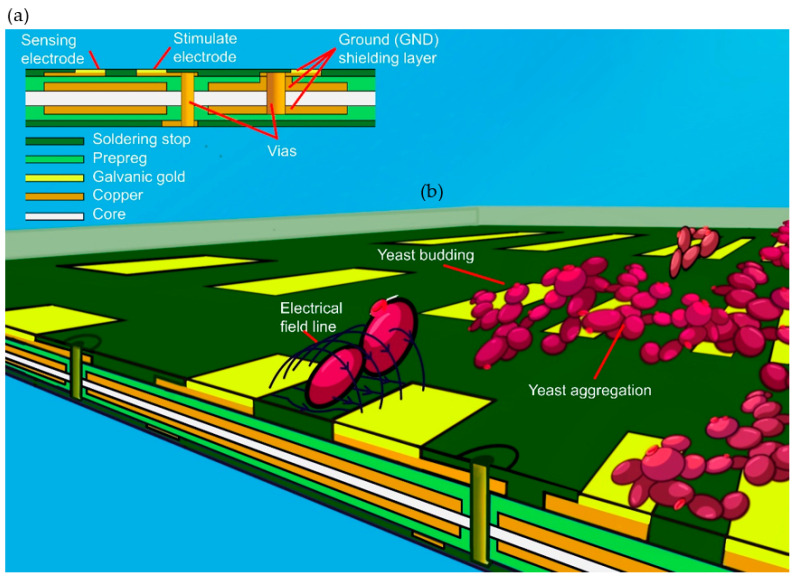
(**a**) 3D illustration of the MEA in four-layer technology. (**b**) Impedance imaging of cells suspended in a solution. Co-planar electrodes were distributed evenly in a 20 × 20 mm rectangular area to form an 8 × 8 array of electrode pairs. A quadratic barrier with a height of 5 mm was bonded to the PCB to establish a liquid reservoir above the MEA for the cell suspension. The co-planer electrodes are overcoated with electroplated gold. Each electrode covers an area of 1.5 × 0.6 mm. The two electrodes for one image point are separated by 0.3 mm.

The MEA with 8 × 8 coplanar electrode pairs was realized based on a commercial low-cost PCB four-layer technology (Multi Leiterplatten GmbH, Brunnthal, Germany) with layers for shielding, interconnection, and electrodes as illustrated in Figure 2.

The measured impedance value of every electrode pair indicates one pixel value in the mapping and the concentration of the cells are scaled as color range from red (high concentration) to blue (low concentration).

### 2.2. Setup for Data and Image Acquisition

A 3D printed barrier with a height of 5 mm was adhesively bonded to the PCB to surround the MEA. Into the resulting reservoir, 4.5 mL of the solution was accurately dispensed with a pipette to ensure that the liquid level was reproducible. Commercial dried edible yeast powder (OSNA Nährmittel GmbH, 12 months before the expiry date) was mixed with sugar (sucrose, Nordzucker AG) water (3 g/L) to prepare different concentrations of yeast suspensions. While the impedances were measured, the CCD camera directly above the device recorded the spreading of cells optically. 

**Figure 3:**
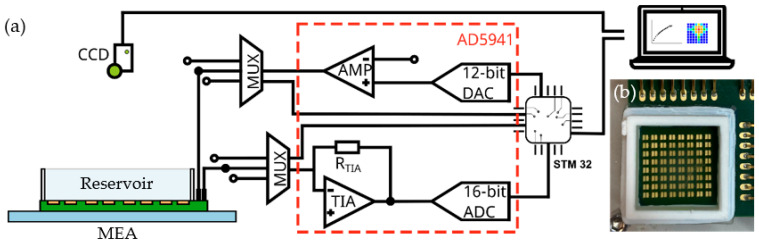
(**a**) Schematic illustration of the experimental setup with impedance readout circuitry connected to the MEA forming the bottom of a liquid reservoir. (**b**) Photograph of 8 × 8 microelectrode array (16 mm × 17 mm) with 3D printed reservoir walls.

**Figure 4:**
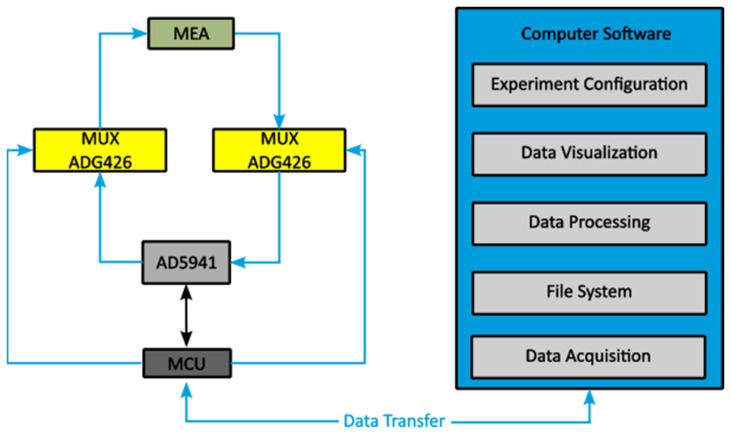
Function block diagram of the impedance measurement platform. The electrode array (MEA) was connected via two multiplexers (ADG426). The microcontroller (STM32H743) controlled data acquisition and activated stimulus signals from the impedance front end (AD5941). Different functional blocks including data processing and visualization routines were integrated in the home-made software which communicates with the microcontroller.

A read-out circuitry was built with commercial components as sketched in Figure 3. With the high precision impedance and electrochemical front end (AD5941 from Analog Devices) stimulus signals were generated by a wave generator and the current was amplified via the transimpedance amplifier (TIA) which can handle signals up to 200 kHz. The AD5941 can perform fast Fourier transformation (FFT) calculation via a hardware accelerator on the chip, which increases the speed of magnitude and phase data of acquisition. The impedance information was sent via serial peripheral interface (SPI) to the microcontroller unit (MCU, STM32H743 from STMicroelectronics). As sketched in Figure 4, the PC could give commands and retrieve data through the MCU to start cyclic voltammetry (CV), electrical impedance spectroscopy (EIS), and single frequency measurement tasks. A software was designed to form the impedance images on the PC. Each electrode pair produced one image point with an impedance value. With the help of two high performance multiplexers (ADG426 from Analog Devices), 64 impedance values were obtained by sequentially scanning rows and columns, which were entered via the serial interface into the software to generate 8 × 8 pixel images. The software was able to configure measurement procedures and to visualize measurement results in different forms.

## 3. Results

### 3.1. Selection of MEA Excitation Conditions

**Figure 5:**
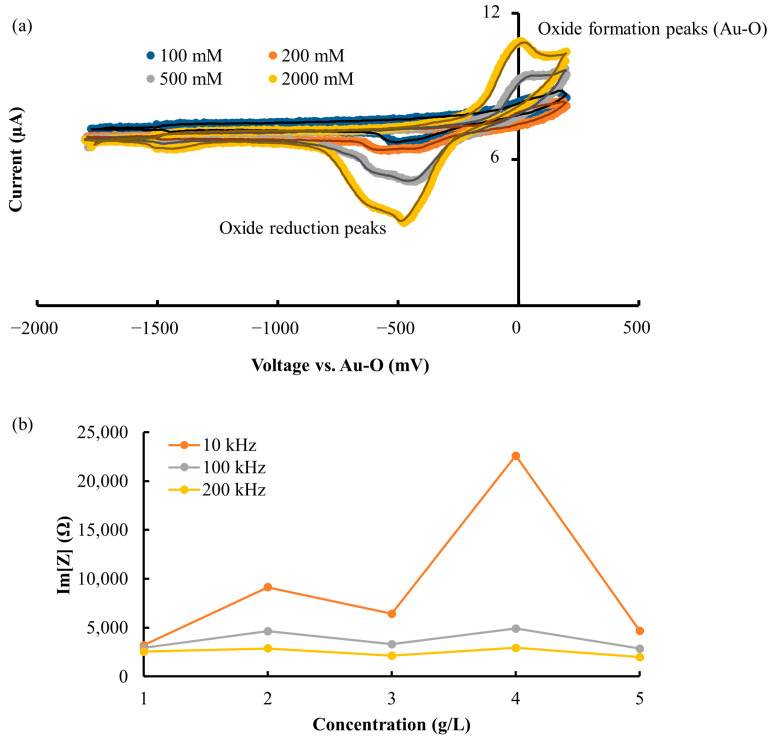
(**a**) The CV curves at varied salt concentrations (no sugar added). The Au-O oxide formation is set as reference (zero point of voltage scale). Electrochemical activity even for higher concentrations is only observed above −1000 mV and below −300 mV vs. Au-O, which indicates a window in which undesirable electrochemical processes can be avoided. (**b**) Im[Z] at frequency of 10 kHz, 100 kHz, and 200 kHz measured at voltage of −1000 mV vs. Au-O for varied sugar concentrations (no salt added), which shows that measurements at higher frequency can avoid undesired influences from sugar.

For the selection of good operational conditions, measurements with a single micro electrode pair were performed. The strength of stimulation signals must be adapted to the composition of the sample. Then, 100 mM, 200 mM, 500 mM, and 2000 mM NaCl solutions were filled into the reservoir. For a good signal–noise ratio, the value of the voltage must be sufficient. However, too high voltage leads to undesirable electrochemical reactions, such as hydrolysis. Cyclic voltammetry measurements of the solutions were performed by applying isosceles triangle wave voltage stimulus and measuring the current response with the described two Au-electrodes system. CV measurements are presented in Figure 5a and show that the characteristic features of Au electrode which the oxide reduction peaks (between −300 and −800 mV vs. Au-O) and oxides formation peaks (between −200 and 200 mV vs. Au-O) diminish with decreasing ion concentration. For all subsequent experiments, −1000 mV vs. Au-O was chosen as a safe stimulus voltage amplitude in which electrochemical reactions using gold electrodes can be prevented. CV results are in agreement with other studies on Au electrodes [20,21,22,23]. In a next step, solutions of 1 g/L to 5 g/L of sugar in DI water were measured at 10 kHz, 100 kHz, and 200 kHz. Values of the imaginary part of impedance Im[Z] are shown in Figure 5b as function of sugar concentration. As the frequency increases, Im[Z] becomes more independent of sugar concentration. The solution of DI water and sucrose is of pure polar nature and will have three different types of interactions at the molecular level, including the water–water, sucrose–sucrose, and water–sucrose dipoles which finally determine the overall dielectric behavior of the solution. A description and explanation of this complex behavior can be found in the literature [24]. The impedance behavior of sucrose solution as we observed (with dips) is very similar to that described there. The similar impedance behavior of the sucrose solution is also shown in another study [24]. Because at 200 kHz independence of sugar concentration in the considered concentration range was practically reached, this frequency was later used in the impedance imaging.

**Figure 6:**
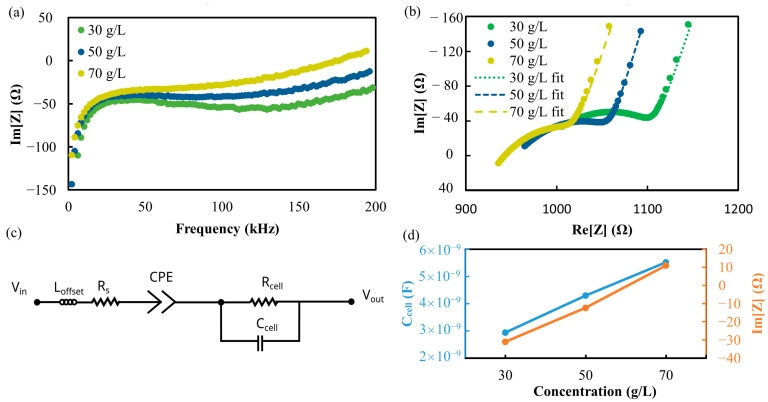
Different yeast cell concentrations measured with a single microelectrode pair. Im[Z] as function of frequency (between 4 kHz and 200 kHz) shows a clear dependence on dry mass of yeast added to water (**a**). Nyquist plot of the EIS measurements (same frequency range) at different cell concentrations with fitted curves (**b**) assuming equivalent circuit (**c**). Fit values for C_cell_ and Im[Z] in dependence of cell concentration (**d**).

In a third step, impedance spectra of different yeast concentrations within 1 g/L sugar solution in normal water, 30 g/L, 50 g/L, and 70 g/L were recorded as shown in Figure 6a. The differences are more pronounced at higher frequencies, in accordance with the expected onset of β-dispersion. To obtain equivalent circuit parameters, the data were plotted as Nyquist diagrams in Figure 6b and fitted by ZView (Scribner Associates Inc.). As sketched in Figure 6c, an equivalent circuit was assumed including a solution resistance (R_s_), a constant–phase-element (CPE) representing the double layer on the electrodes, a resistor (R_cell_) connected in parallel with a capacitor (C_cell_), and a small inductive contribution from the measuring electronics (L_offset_) which caused the shift in the Nyquist diagram. R_cell_‖C_cell_ represented the contribution of the cells. 

CPE consists of a capacitance value CPE and a dimensionless exponent CPE-P, which is describing the non-ideality of the double layer capacitance (1 for an ideal capacitance, but usually between 0.8 and 0.9). As can be seen from the fitted parameters in Table 1, the two values for the CPE, L_offset_ and R_s_, are practically insensitive to cell concentration. However, C_cell_ has a strong and monotone dependence of the cell concentration just like Im[Z] measured at 200 kHz (Figure 6d). The latter value does not require any fitting to spectra for its determination, but only one measurement at a fixed frequency. These results indicate that cell concentrations can be obtained with the MEA using 200 kHz sine excitation at an amplitude of −1000 mV vs. Au-O. 

### 3.2. Image Deblurring

**Figure 7:**
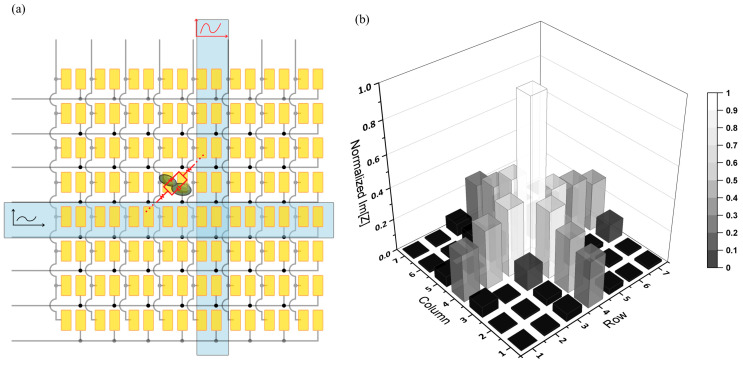
Schematic illustration of the row/column addressing in the MEA (**a**). As an example, cells at row 4–column 4 also contribute to the signal that is obtained when row 5 and column 5 are addressed because of a parasitic current (red). (**b**) Three-dimensional representation of normalized PSF as measured for cells concentrated at the row 5–column 5 electrode pair.

Figure 7a illustrates the row and column addressing in the impedance camera and the occurrence of currents not only between the paired microelectrodes, but also between microelectrodes belonging to different pairs. This means that the signal obtained at a certain pixel position is overlaid by somewhat weaker parasitic influences which lead to a blurring of the image. This blur can be described by a point spread function (PSF) which decreases symmetrically from the point center in the orthogonal row/column directions but in non-orthogonal directions decreases much faster. A discrete PSF for Im[Z] imaging as shown in Figure 7b was obtained by imaging a drop of 20 ul of 70 g/L yeast solution concentrated at the row 5–column 5 electrode pair.

**Figure 8:**
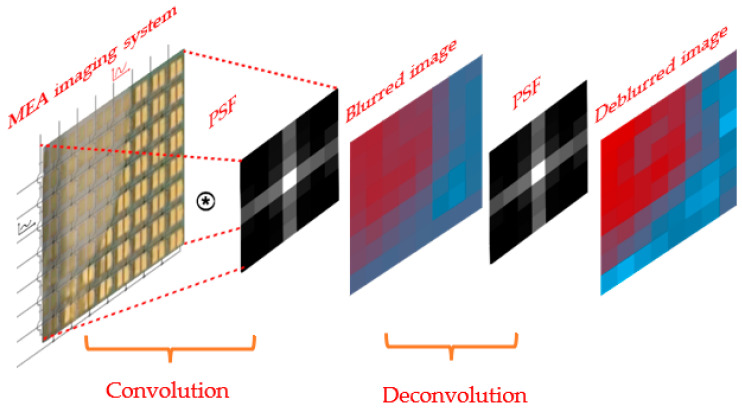
Sketch illustrating the blurring in the impedance camera as convolution with a PSF. Image deblurring is based on a deconvolution algorithm where a PSF obtained from experiment is used as the kernel.

Since those cells located on top of one pair of electrodes create an image point that is blurred by this PSF, the unprocessed image is a convolution of the real distribution of cells with the PSF (see Figure 8). For deblurring, the images were therefore deconvoluted with the PSF as kernel (using MATLAB from The MathWorks, Inc., Natick, MA, USA) as illustrated in Figure 8.

### 3.3. Impedance Imaging

**Figure 9:**
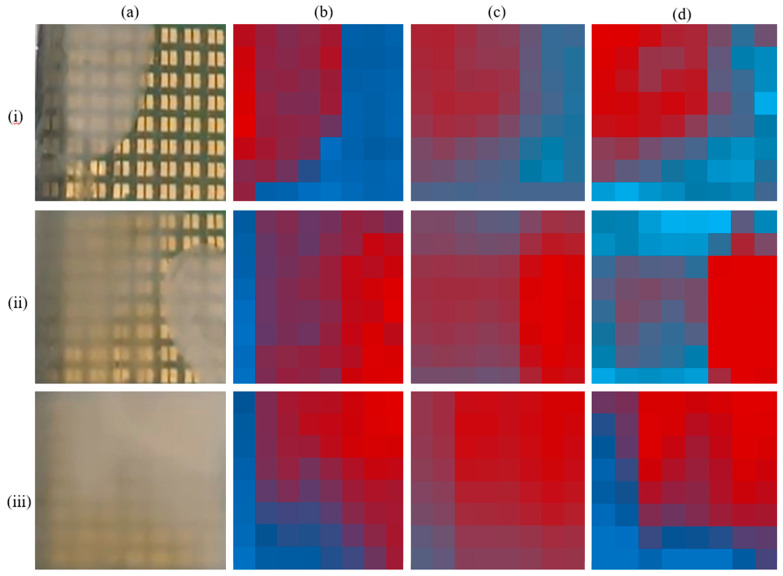
Optical camera images of yeast suspensions with a concentration of 70 g/L dispensed into the reservoir above the MEA to form an inhomogeneous distribution (**a**). Images converted into 8 × 8 pixel image in which each pixel represents the average light intensity calibrated to cover the full range from red = 0 to blue = 255 (**b**). Im[Z] images as obtained with impedance camera after background subtraction (stimulation with 200 kHz at −10 mV amplitude) with same color scale for each image (**c**). Respective Im[Z] images obtained after deblurring (d). Images were obtained one minute after 20 μL solution injected in the upper left corner of the MEA (i), after another 20 μL injected in the lower right corner of the MEA one minute later (ii), and after another 20 μL injected in the upper right corner of the MEA one more minute later (iii). Pixels in (**c**,**d**) represent values of the imaginary part of impedance re-calibrated to cover the full scale from red = 0 to blue = 255.

As a first step, 45 mL of water of 3 g/L sugar solution was added to the reservoir and one Im[Z] imaging was performed to determine the background signals. Then, 20 µL of 70 g/L yeast solution was dispensed into the reservoir at different positions before the Im[Z] imaging was performed 30 s, 2 min 43 s, and 5 min 38 s after dispensing (each time requiring 30 s for scanning and mapping, see video S1 in the Supplementary Materials). After background subtraction, the Im[Z] images were compared with the optical images recorded simultaneously by the optical camera and also with the converted 8 × 8 pixel images representing local averaged light intensities (see Figure 9a,b). Im[Z] images shown in Figure 9c were deblurred based on the experimental PSF (see Figure 9c). As can be seen, the Im[Z] images correlate quite well with the yeast cell distributions in the optical images. A well-known objective image quality metric, thfe peak-signal-to-noise ratio (PSNR), was used to analyze the performance of the deblurring methods. The mean-square-error (MSE) a test image (Img) against a reference image (ref) is calculated as given in Equation (1) assuming that both images are of size of M × N.

(1) $$\mathrm{MSE}\left(\mathrm{ref},\mathrm{Img}\right)=\frac{1}{\mathrm{MN}}\;\overset{M}{\underset{i=1}{\sum }}\overset{N}{\underset{j=1}{\sum }}{\left({\mathrm{ref}}_{\mathrm{ij}}+{\mathrm{Img}}_{\mathrm{ij}}\right)}^{2}$$

The reference images (ref) were taken from Figure 9b and test images (Img) from Figure 9c,d. The PSNR (in dB) assuming an intensity range from 0 to 255 is defined by Equation (2).

(2) $$\mathrm{PSNR}\left(\mathrm{ref},\mathrm{Img}\right)=10\log_{10}\left(255^{2}/\mathrm{MSE}\left(\mathrm{ref},\mathrm{Img}\right)\right)$$

The larger the PSNR value, the better the image quality compared to the reference image. Usually, a value higher than 10 dB means that the structural similarity between reference and test images can be recognized by human eyes [25]. As can be seen in Table 2, deblurring improved the image quality in all three cases (i,ii,iii). However, the improvement for case (iii) is much clearer that for the other cases.

## 4. Discussion

We have successfully developed a low-cost impedance camera that can map the concentration of yeast suspension cells using an MEA obtained by standard four-layer PCB technology and a home-built read-out circuitry made from commercial components. Conditions for amplitude and frequency of the excitation signals were chosen not to induce electrochemical reactions and to provide high sensitivity to cell concentration and negligible sensitivity to ion concentrations in the media. Next to the hardware, a software was developed to control the hardware and to generate the impedance camera images. A deblurring based on an experimentally obtained PSF proved to sharpen the impedance images. The metric PSNR was used to evaluate the impedance image qualities and to prove the success of the deblurring.

Further miniaturization also facilitating portability of the impedance camera technology is possible. Scanning speed can be accelerated but real-time scanning may require more complex electronic systems. Higher resolution can in future be realized with smaller and more electrode pairs, which increases the complexity of the MEA. The camera concept shall in future be integrated into organ-on-chip devices to augment or even replace optical imaging, especially in permeation chips with porous membranes.

## Figures and Tables

**Table 1:** Results of fitting the equivalent circuit given in Figure 5c to the measured Nyquist diagrams as given in Figure 6b together with respective root mean square error (RMSE), which is the standard deviation of the residuals (not of the measurement values).

	*L_offset_* (nH)	*R_s_* (Ω)	CPE (µF)	CPE-P	*C_cell_* (µF)	*R_cell_* (Ω)
**30 g/L**	7 × 10^−5^	900	2 × 10^−6^	0.8	2.9 × 10^−9^	228
**RMSE**	5 × 10^−7^	0.4	8 × 10^−8^	0.004	9.7 × 10^−11^	2.2
**50 g/L**	7 × 10^−5^	920	2 × 10^−6^	0.8	4.3 × 10^−9^	182
**RMSE**	8 × 10^−7^	2.2	4 × 10^−8^	0.002	9.3 × 10^−11^	2.1
**70 g/L**	7 × 10^−5^	860	2 × 10^−6^	0.8	5.4 × 10^−9^	151
**RMSE**	2 × 10^−7^	0.2	4 × 10^−8^	0.002	0.1 × 10^−9^	0.3

**Table 2:** Peak-signal-to-noise ratio (PSNR) as objective image quality metrics for analyses of blurred/deblurred images.

PSNR (ref, Img) (dB)	(c)	(d)
**(i)**	13.7	14.9
**(ii)**	11.3	13.2
**(iii)**	6.1	16.0

## Data Availability

Not applicable.

## Supplementary Materials

The following supporting information can be downloaded at: https://www.mdpi.com/article/10.3390/bios13020281/s1, Video S1: Yeast cell distribution.
